# Evaluation of Genotoxic and Cytotoxic Effects in Human Peripheral Blood Lymphocytes Exposed *In Vitro* to Neonicotinoid Insecticides News

**DOI:** 10.1155/2012/612647

**Published:** 2012-03-27

**Authors:** María Elena Calderón-Segura, Sandra Gómez-Arroyo, Rafael Villalobos-Pietrini, Carmen Martínez-Valenzuela, Yolanda Carbajal-López, María del Carmen Calderón-Ezquerro, Josefina Cortés-Eslava, Rocío García-Martínez, Diana Flores-Ramírez, María Isabel Rodríguez-Romero, Patricia Méndez-Pérez, Enrique Bañuelos-Ruíz

**Affiliations:** ^1^Laboratorios de Citogenética y Mutagénesis Ambientales, Centro de Ciencias de la Atmósfera, Universidad Nacional Autónoma de México, Ciudad Universitaria Coyoacán, 04510 México city, DF, Mexico; ^2^Departamento de Ciencias Biológicas, Universidad de Occidente, Boulevard Macario Gaxiola, Carretera Internacional, Los Mochis, SIN, 81223 México, Mexico; ^3^Unidad Académica de Ciencias Químico Biológicas, Avenida Lázaro Cárdenas S/N, Universidad Autónoma de Guerrero, Ciudad Universitaria, Chilpancingo, GRO, 39090 México, Mexico; ^4^Laboratorio de Química Atmosférica, Centro de Ciencias de la Atmósfera, Universidad Nacional Autónoma de México, Ciudad Universitaria Coyoacán, 04510 México, Mexico

## Abstract

Calypso (thiacloprid), Poncho (clothianidin), Gaucho (imidacloprid), and Jade (imidacloprid) are commercial neonicotinoid insecticides, a new class of agrochemicals in México. However, genotoxic and cytotoxic studies have not been performed. In the present study, human peripheral blood lymphocytes (PBL) were exposed *in vitro* to different concentrations of the four insecticides. The genotoxic and cytotoxic effects were evaluated using the alkaline comet and trypan blue dye exclusion assays. DNA damage was evaluated using two genotoxicity parameters: tail length and comet frequency. Exposure to 9.5 × 10^−6^ to 5.7 × 10^−5^ M Jade; 2.8 × 10^−4^ to 1.7 × 10^−3^ M Gaucho; 0.6 × 10^−1^ to 1.4 × 10^−1^ M Calypso; 1.2 × 10^−1^ to 9.5 × 10^−1^ M Poncho for 2 h induced a significant increase DNA damage with a concentration-dependent relationship. Jade was the most genotoxic of the four insecticides studied. Cytotoxicity was observed in cells exposed to 18 × 10^−3^ M Jade, 2.0 × 10^−3^ M Gaucho, 2.0 × 10^−1^ M Calypso, 1.07 M Poncho, and cell death occurred at 30 × 10^−3^ M Jade, 3.3 × 10^−3^ M Gaucho, 2.8 × 10^−1^ M Calypso, and 1.42 M Poncho. This study provides the first report of genotoxic and cytotoxic effects in PBL following *in vitro* exposure to commercial neonicotinoid insecticides.

## 1. Introduction

In 2008, Bayer CropScience México marketed a new class of agrochemicals, the neonicotinoids [1]. These insecticides are derivatives of nicotine and are classified as N-nitroguanidines (imidacloprid, thiamethoxam, dinotefuran, and clothianidin) and N-cyano-aminides (acetamiprid and thiacloprid). These active components determine the insecticidal potency and selectivity of the insecticides [2].

A number of commercial formulations of neonicotinoid insecticides have been marketed. Poncho (active ingredient: clothianidin) is a systemic insecticide used to treat corn seeds against insects. Calypso 480 SC (active ingredient: thiacloprid) is applied to the foliage of cotton, apple, potatoes, and ornamental plants to control a number of insects. Two commercial formulations of imidacloprid are used: Gaucho 70 S is used to treat cucumber, pepper, squash, tomato, and tobacco pests, while Jade is used exclusively to control *Aeneolamia postica* in sugarcane crops [1]. These insecticides pose relatively little risk to nontarget organisms and the environment. Additionally, they have a high level of efficacy at low concentrations. As a result of these properties, neonicotinoid pesticides have begun replacing pyrethroids, chlorinated hydrocarbons, organophosphates, carbamates, and other insecticides [3].

Neonicotinoid insecticides are potent selective agonists of the nicotinic acetylcholine receptor (nAChR) in both invertebrates and vertebrates [4–6]. They are classified by the EPA [7] as class II [8, 9] and III toxins and are labeled with a signal word: “Warning” or “Caution” [10]. Thiacloprid and thiamethoxam are likely human carcinogens [7–9]. In contrast, there is no evidence of carcinogenicity for imidacloprid and chlotiniadin [7–9]. Thiacloprid induces ovary tumors in mice [7–9] and uterine tumors in rats [7–9]. Thiamethoxam produces liver tumors in female and male mice [11, 12]. Imidacloprid and thiacloprid decrease reproduction rates in *Caenorhabditis elegans *and *Eisenia fetida* [13]. After metabolic activation *in vitro* (S9 enzymatic mix from rat livers), imidacloprid produces calf thymus DNA adducts [14], increases the frequency of spermatic abnormalities in *Eisenia fetida* [15], and is mutagenic in TA98 and TA100 *Salmonella typhimurium *strains, with or without S9 metabolic activation [16]. Imidacloprid also induces significant increases in the frequency of sister chromatid exchange and micronuclei formation in human peripheral blood lymphocytes [17, 18], mice and rat bone-marrow cells [16, 19], tadpole peripheral blood erythrocytes from *Rana N*-*Hallowell* [20], and *Vicia faba* roots [15]. Furthermore, imidacloprid causes DNA strand breaks in the coelomocytes of *Eisenia fetida* [15], tadpole erythrocytes from *Rana N-Hallowell* [20], human peripheral blood lymphocytes [17], and leukocytes in culture [18]. It does not cause DNA strand breaks in *Vicia faba* roots [21]. Acetamiprid is another insecticide that causes sister chromatid exchange, micronuclei formation, and chromosomal aberrations in human peripheral blood lymphocytes *in vitro *[22].

There are reports of neonicotinoid poisoning (imidacloprid and acetamiprid) in humans [23–27]. It is important to evaluate the genotoxic and cytotoxic actions of these new agricultural pesticides to contribute with toxicological data and regular use without polluting the environment and without leaving their residues in water and food sources with their possible risk on the organism health. But, in Mexico, there are no regulatory agencies on the pesticides safety requirements and lack of knowledge on appropriate protective measures and equipments by agricultural workers that could help them reduce the risk to exposure to agrochemicals.

Mexican agricultural workers are exposed to different pesticide mixtures, including organophosphates, organochlorines, carbamates, and pyrethroids, increase of the incidence of diseases and cancer. Differences in the exposure pathways to pesticides are responsible for variations in the tissue and organ distributions, patterns of biological effects, and toxic potency. The effects of exposure are dependent on diverse factors, including lifestyle, diet, state of chemoprotection, genetic predisposition or polymorphic expression of certain enzymes (e.g., CYP450s), and general health [28, 29].

Many studies have demonstrated that occupational exposure to pesticides induces DNA damage [30], such as sister chromatid exchange [31, 32], micronuclei formation [28, 33], chromosomal aberrations [34, 35], DNA adducts [36], and DNA strand breaks [37]. Numerous epidemiological studies have associated DNA damage with an increased incidence of functional alterations in the nervous [38], respiratory [39], reproductive [40], and immune systems [41, 42]. DNA damage is also associated with an increased risk of cancer [41–46]. DNA lesions could be an initial event in the process of chemical carcinogenesis [47, 48], and the development of tumors may eventually occur [49]. In general, the induction of genotoxicity such as chromosomal and DNA lesions may lead to further problem of mutagenic and carcinogenic activity [49]. DNA damage is the under cause of mutations leading to cancer [47–49].

 Considering that neonicotinoid insecticides are a new class of agrochemicals and there is lack of genotoxic studies on these pesticides, the present study aims to evaluate the genotoxic and cytotoxic effects of these chemicals. Human peripheral blood lymphocytes were exposed to Calypso (thiacloprid), Poncho (clothianidin), Gaucho, and Jade (both imidacloprid), and DNA damage was assessed using the alkaline comet and cell viability assays. The comet assay is a quick, simple, and sensitive method to detect DNA damage (single- and double-strand breaks, alkali-labile sites or DNA-DNA and DNA-protein crosslinks) induced by environmental chemical agents in individual cells, tissues, and organs [50, 51]. This assay has been proven to be reliable for the evaluation of genetic damage induced by agrochemicals both *in vitro* [52] and *in vivo *[37].

## 2. Materials and Methods

### 2.1. Chemicals

Hank's balanced salt solution, phosphate buffered saline, RPMI 1640 medium, normal-melting-point agarose, and penicillin/streptomycin were purchased from GIBCO, Ficoll-PaqueTM PLUS was obtained from GE Healthcare; low-melting-point agarose, trypan blue (0.4%), ethidium bromide, Trizma Base, triton X-100 were purchased from Sigma-Aldrich, and ethylenedinitrilo-tetraacetic acid, and sodium hydroxide, sodium chloride were obtained from Baker.

### 2.2. Preparation of the Neonicotinoid Insecticide Concentrations

**Figure 1:**
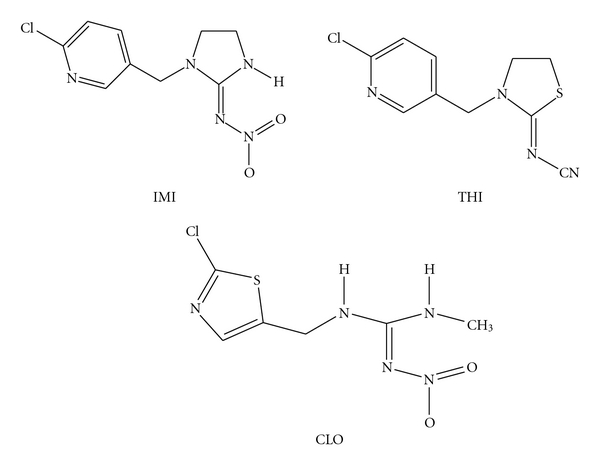
Chemical structures of imidacloprid (IMI), thiacloprid (THI) and clothianidin (CLO).

The neonicotinoid insecticides were donated by Bayer Cropscience (México) [1]. For the genotoxic and cytotoxic assays (Figure 1), Calypso 480 SC ({(2*Z*)-3-[(6-chloropyridin-3-yl)methyl]-1,3-thiazolidin-2-ylidene} cyanamide; flowable suspension: thiacloprid, 480 g a.i./L; RSCO-INAC-0102T-301-064-040), Poncho (*E*-1-(2-chloro-1,3-thiazol-5-ylmethyl)-3-methyl-2-nitroguanidine; RSCO-INAC-103K-301-342-048; flowable suspension: clothianidin, 600 g a.i/L), and Gaucho 70WS (*N*-[1-[(6-chloro-3-pyridyl)methyl]-4,5-dihydroimidaclopriddazol-2-yl]nitramide; dispersible powder: imidacloprid, 700 g a.i./L; RSCO-INAC-0199-305-034-070) were diluted 1 : 10 mL with deionized water, while Jade (granule: imidacloprid, 8 g a.i/Kg) was diluted 3 : 10 in deionized water.

### 2.3. Isolation of Lymphocytes from Human Peripheral Blood

 Twenty milliliters of heparinized venous blood obtained from three healthy volunteer donors was centrifuged at 2,500 rpm for 20 min. The cellular layer was diluted 1 : 1 with HBSS, placed over a Ficoll-Paque layer, and centrifuged at 1,500 rpm for 10 min. Lymphocytes were collected and washed twice in RPMI 1640 medium by centrifugation at 1,500 rpm for 10 min. The lymphocyte pellet was kept in RPMI 1640 medium (37°C) supplemented with 1% penicillin/streptomycin and immediately assessed for changes in cellular viability using a Neubauer chamber.

### 2.4. Cell Viability Test

 Cell viability was estimated before and after treatments using the trypan blue exclusion method [53]. Trypan blue penetrates the damaged membrane of dead cells and stains the nucleus. A mix of 10 *μ*L of cell pellet and 10 *μ*L of trypan blue was incubated for 3 min. Then the number of dead cells out of 100 consecutive cells was counted in duplicate.

### 2.5. Neonicotinoid Insecticide Treatment of Human Peripheral Blood Lymphocytes In Vitro

Human peripheral blood lymphocytes (5 × 10^5^ cells) with a viability >92% were incubated with 9.5 × 10^−6^, 1.9 × 10^−5^, 2.8 × 10^−5^, 3.8 × 10^−5^and 5.7 × 10^−5^ M Jade; 2.8 × 10^−4^, 5.7 × 10^−4^, 8.3 × 10^−4^, 1.1 × 10^−3^and 1.7 × 10^−3^ M Gaucho; 0.6 × 10^−1^, 0.9 × 10^−1^, 1.2 × 10^−1^, 1.3 × 10^−1^ and 1.4 × 10^−1^ M Calypso; 1.2 × 10^−1^, 2.4 × 10^−1^, 4.8 × 10^−1^, 7.1 × 10^−1^ and 9.5 × 10^−1^ M Poncho in 1 mL of 1640 RPMI medium at 37°C for 2 h. The controls consisted of human peripheral lymphocytes (5 × 10^5^ cells) in RPMI 1640 medium under the same conditions. After treatments, the cells were washed twice with RPMI 1640 medium and subjected immediately to the cell viability and alkaline comet assays.

### 2.6. Alkaline Comet Assay

**Figure 2:**
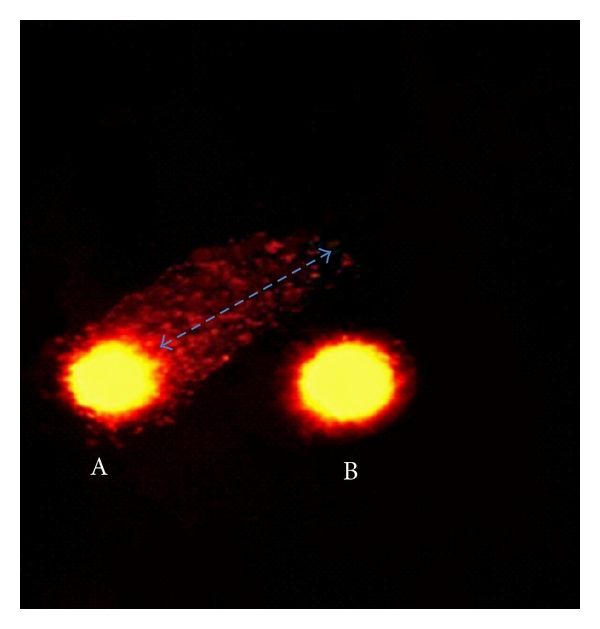
Human peripheral blood lymphocytes. A: nuclei with DNA damage (with comet) and B: nuclei without DNA damage (without comet).

The alkaline comet assay was performed according to procedures previously described by [50, 51]. Briefly, lymphocytes (2,500 cells) were mixed with 90 *μ*L of low-melting-point agarose (0.5%) at 37°C, placed on fully frosted slides (Fisher) coated with a thin layer of normal-melting-point agarose (1%) and covered with a coverslip. Two slides were made for each treatment. The slides were kept at 4°C for 5 min to allow the agarose to solidify. The coverslip was then carefully removed, and the slides were immersed in a Coplin staining jar containing a freshly prepared cold lysis solution (2.5 M NaCl, 100 mM EDTA, 10 mM Tris, 1% Triton X-100, and 10% DMSO, pH = 10) at 4°C for 1 h. The slides were placed in a horizontal electrophoresis chamber (Owl A5, Lab System Inc) containing freshly prepared cold electrophoresis alkaline buffer (300 mM NaOH, 1 mM EDTA, pH = 13) for 20 min to unwind the DNA. Electrophoresis was carried out at 25 V and 300 mA for 20 min in darkness to prevent additional DNA damage. The slides were then washed three times with freshly prepared neutralization buffer (0.4 M Tris, pH 7.5) for 5 min, fixed with cold absolute methanol for 5 min, and air-dried at room temperature. Next, 50 *μ*L of ethidium bromide (20 mg/mL) was added to each slide to stain the DNA. The slides were labeled with a code that was unfamiliar to the viewer and examined with an Axiostar Plus Carl Zeiss fluorescent microscope equipped with an excitation filter (515–560 nm) and a barrier filter (590 nm). To visualize DNA damage, slides were observed at 40x magnification using a micrometric eyepiece/objective combination (1 unit = 2.41 *μ*m at 40x magnification). Two parameters were used to determine genotoxicity: (a) comet frequency (nuclei with DNA damage) in 50 randomly selected nuclei on each slide (two slides per treatment); and (b) comet tail length (DNA fragmentation), evaluated by measuring the distance (in *μ*m) from the nuclear region to the end of the tail in 100 consecutive nuclei (Figure 2A).

### 2.7. Statistical Analysis

Comet frequency, tail length, and cell viability are reported as the mean ± standard error of the mean (SEM) obtained from three independent experiments for each treatment. An analysis of variance (ANOVA) and the Newman-Keuls test were used to determine significant differences between the treatment groups. Significance was defined as *P* < 0.001. The relationship between comet frequency and comet tail length was evaluated using linear regression analysis.

## 3. Results

### 3.1. In Vitro Genotoxicity of Neonicotinoid Insecticides in Human Peripheral Blood Lymphocytes

**Figure 3:**
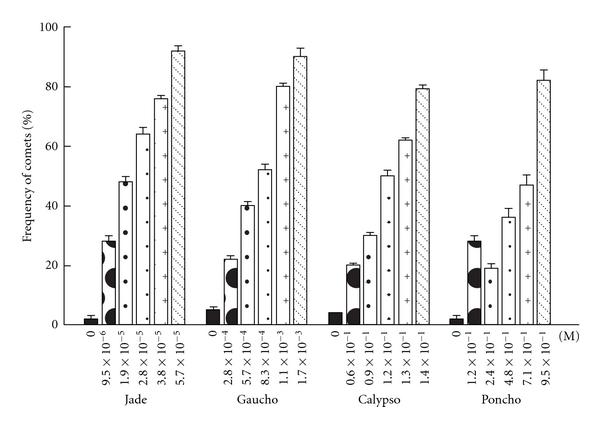
Mean frequency of nuclei with comet in human peripheral blood lymphocytes exposed *in vitro* to neonicotinoid insecticides. The bars represent the mean values ± SEM of the comet from the three independent experiments.

**Figure 4:**
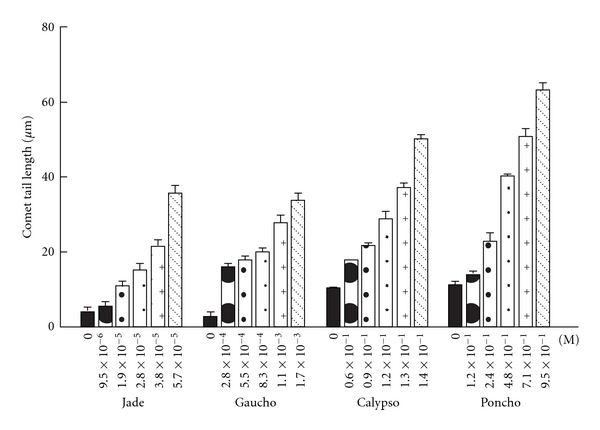
Mean comet tail length in human peripheral blood lymphocytes exposed *in vitro* to neonicotinoid insecticides. The bars represent the mean values ± SEM of the comet tail length from the three independent experiments.

Exposure to all concentrations of Calypso 480 SC (thiacloprid), Poncho (Clothianidin), and Gaucho and Jade (two imidacloprid commercial formulations) for 2 h caused significant increases in the two measures of genotoxicity, percentage of comets and the tail length, in relation to the controls (*P* < 0.001; Figures 3 and 4). In cells exposed to 0.6 × 10^−1^, 0.9 × 10^−1^, 1.2 × 10^−1^, 1.3 × 10^−1^, and 1.4 × 10^−1^ M Calypso, the mean comet frequency ranged from  20 ± 0.6  to  79 ± 0.7  and the tail length ranged from 17.9   ±   0.1 to 50.3 ± 1.0 *μ*m. In cells exposed to 1.2 × 10^−1^, 2.4 × 10^−1^, 4.8 × 10^−1^, 7.1 × 10^−1^, and 9.5 × 10^−1^ M Poncho, the mean comet frequency ranged from  7 ± 0.2  to  82 ± 3.4  and the tail length ranged from 14.2 ± 0.8 to 63.3 ± 2.0 *μ*m. In cells exposed 9.5 × 10^−6^, 1.9 × 10^−5^, 2.8 × 10^−5^, 3.8 × 10^−5^ and 5.7 × 10^−5^ M Jade, the mean comet frequency ranged from  28 ± 2.0  to  92 ± 1.7  and the tail length ranged from 5.5 ± 1.1 to 35.6 ± 2.2 *μ*m. In cells exposed to 2.8 × 10^−4^, 5.7 × 10^−4^, 8.3 × 10^−4^, 1.1 × 10^−3^ and 1.7 × 10^−3^ M Gaucho, the mean comet frequency ranged from  22 ± 1.2  to  90 ± 2.8  and the tail length ranged from 15.71 ± 1.2 to 33.94 ± 1.9 *μ*m (Figures 3 and 4). At the highest concentrations, all neonicotinoid insecticides caused severe DNA damage in 80–90% of nuclei, exhibiting a higher comet frequency and greater tail length when compared to controls (Figures 3 and 4).

The relative genotoxicities of neonicotinoid insecticides in human peripheral blood lymphocytes *in vitro *are as follows: Jade > Gaucho > Calypso > Poncho (Figures 3 and 4). Control lymphocytes from three healthy volunteer donors showed low basal DNA damage (Figures 3 and 4).

**Figure 5:**
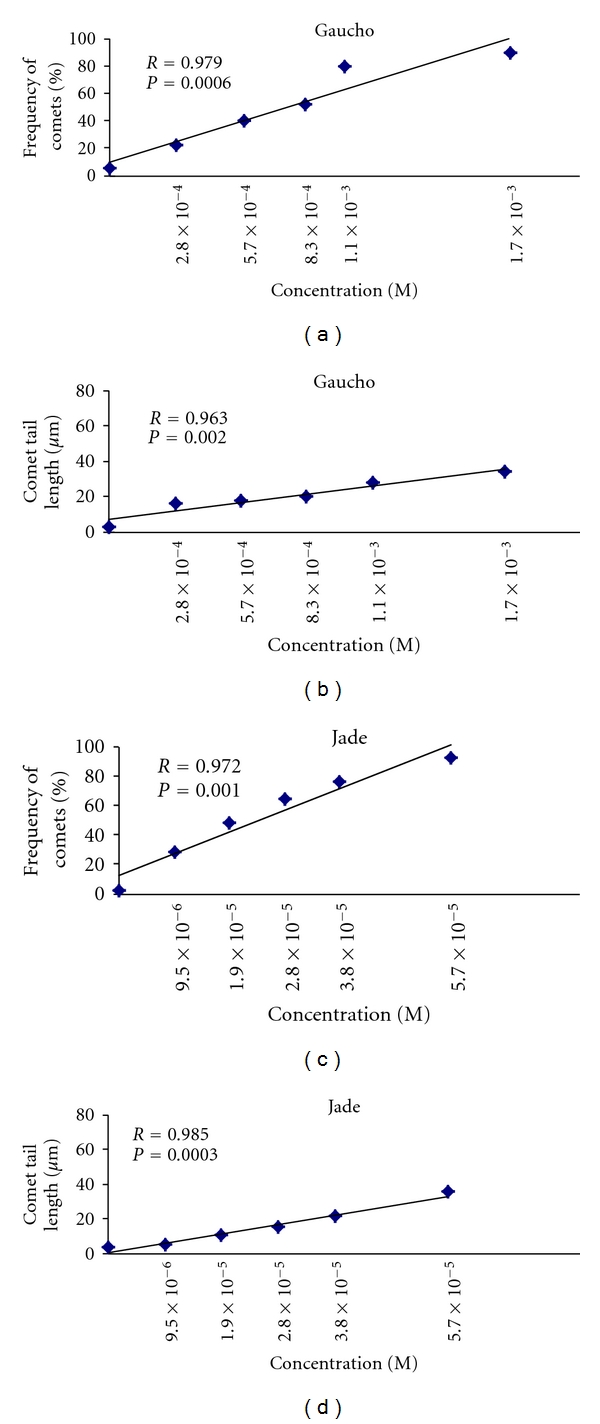
Regression lines of the frequency of comets and comet tail length in human peripheral blood lymphocytes exposed *in vitro* to Gaucho and Jade.

**Figure 6:**
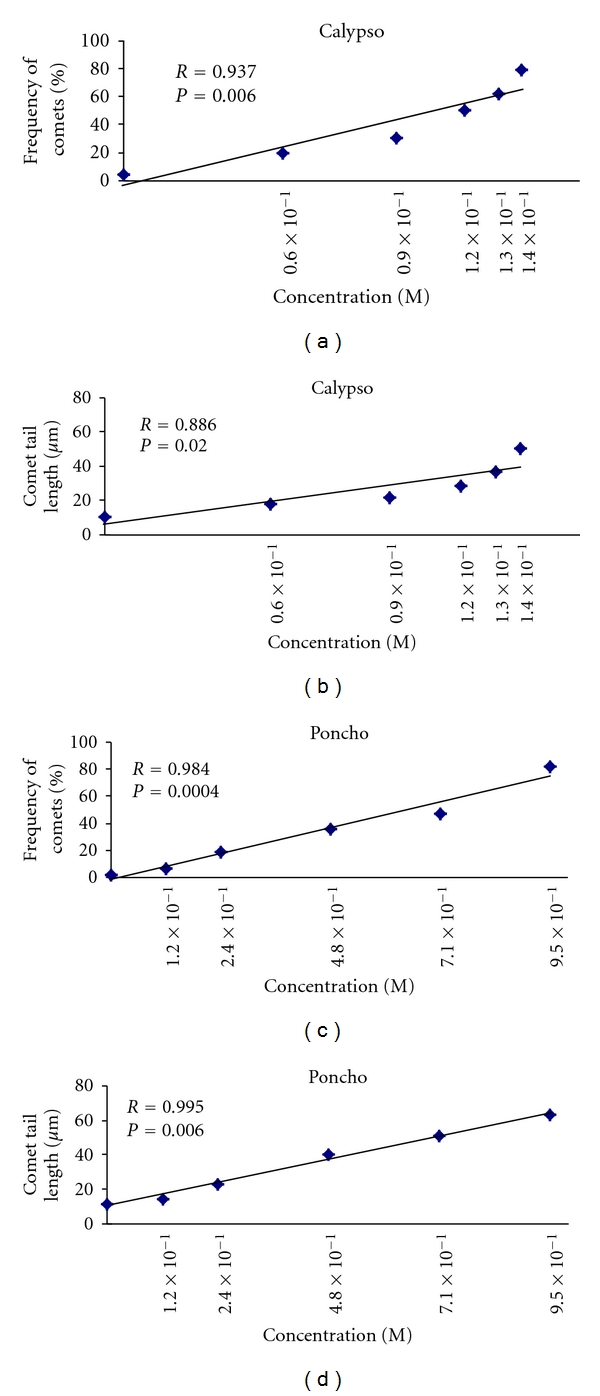
Regression lines of the frequency of comets and comet tail length in human peripheral blood lymphocytes exposed *in vitro* to Calypso and Poncho.

The linear regression analysis of the mean comet frequency and the comet tail length in all neonicotinoid insecticides showed positive correlations (*r* = 0.9), indicating a concentration-dependent relationship (Figures 5 and 6).

### 3.2. In Vitro Cytotoxicity of Neonicotinoid Insecticides in Human Peripheral Blood Lymphocytes

**Figure 7:**
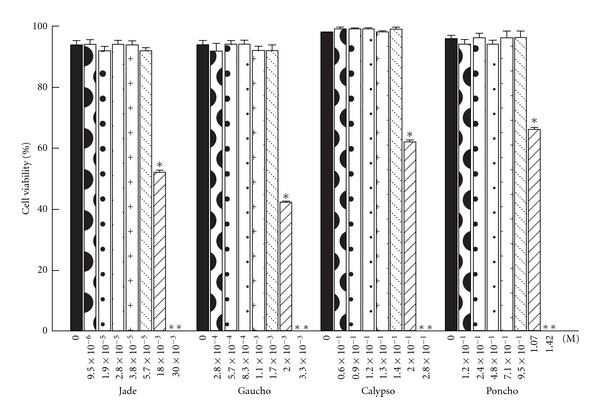
Mean viability of the human peripheral blood lymphocytes exposed *in vitro* to neonicotinoid insecticides. The bars represent the mean values ± SEM from three independent experiments. *indicates significant decrease in cell viability; **indicates cell death.

 In the preliminary experiments, human peripheral blood lymphocytes were exposed to different concentrations of four neonicotinoid insecticides for 2 h. After treatment, cell viability was evaluated by trypan blue dye-exclusion staining. The data indicate that concentrations of 9.5 × 10^−6^, 1.9 × 10^−5^, 2.8 × 10^−5^, 3.8 × 10^−5^, and 5.7 × 10^−5^ M Jade; 2.8 × 10^−4^, 5.7 × 10^−4^, 8.3 × 10^−4^, 1.1 × 10^−3^and 1.7 × 10^−3^ M Gaucho; 0.6 × 10^−1^, 0.9 × 10^−1^, 1.2 × 10^−1^, 1.3 × 10^−1^ and 1.4 × 10^−1^ M Calypso; and 1.2 × 10^−1^, 2.4 × 10^−1^, 4.8 × 10^−1^, 7.1 × 10^−1^ and 9.5 × 10^−1^ M Poncho did not produce statistically significant differences in cell viability when compared to controls (*P* < 0.001; Figure 7). These concentrations were then used for the alkaline comet assay. However, when the human lymphocytes were exposed to 18 × 10^−3^ M Jade, 2.0 × 10^−3^ M Gaucho, 2.0 × 10^−1^ M Calypso, and 1.07 M Poncho, cell viability was significantly decreased in relation to the control values (*P* < 0.001). Cell death occurred following exposure to 30 × 10^−3^ M Jade, 3.3 × 10^−3^ M Gaucho, 2.8 × 10^−1^ M Calypso, and 1.42 M Poncho (Figure 7).

## 4. Discussion

Neonicotinoid pesticides represent 17% of all processed insecticides on the global market [54]. This is largely because they are less persistent in the environment, do not accumulate in animal tissues, and are less toxic to mammals than older classes of insecticides. Neonicotinoids, therefore, represent a new and “less hazardous” class of agrochemicals [2, 55, 56]. However, until now, no studies have been performed to evaluate their genotoxic and cytotoxic effects. The present study evaluated the genotoxic and cytotoxic effects of the neonicotinoid insecticides Calypso, Poncho, Gaucho, and Jade in human peripheral blood lymphocytes *in vitro *using the alkaline comet and trypan blue exclusion assays. The alkaline comet assay is an early biomarker that is widely used to detect DNA damage induced by environmental chemical agents, such as pesticides. When compared with other cytogenetic tests, such as sister chromatid exchange, micronuclei formation, and chromosomal aberration assays, the comet assay is the most rapid and sensitive method to evaluate the genotoxic agents both *in vitro* and *in vivo*; this assay does not require proliferation of cells, is applicable to all eukaryotic cells, and can obtain reproducible results in a very short amount of time [51].

Low concentrations of all the tested neonicotinoid pesticides induced DNA damage, resulting in significant increases in the two measures of genotoxicity used in the present study: comet frequency and tail length. Our results are in agreement with data obtained from *in vitro* genotoxic studies performed with the insecticide imidacloprid. Exposure to 1 mM Admire that was metabolically activated *in vitro* with a rat liver S9 enzymatic mixture produced calf thymus DNA adducts [14]. Concentrations of 25–100 *μ*L/plaque Confidor significantly augmented the reverse mutation rate of TA 98 and TA 100 *Salmonella typhimurium* strains, both with and without *in vitro* S9 metabolic activation (S9 enzymatic mix from rat livers) [16]. In culture, human peripheral blood lymphocytes exposed to 0.1 or 0.5 mg/L imidacloprid (pure compound) showed significantly increased levels of sister chromatid exchange and micronuclei formation [17], and 0.05, 0.1, 0.2, and 0.5 mg/L imidacloprid enhanced DNA strand breaks [17]. However, 0.1, 1, 5, 50, and 100 *μ*g/mL imidacloprid incubated with the same human cells *in vitro* were negative for sister chromatid exchange and micronuclei formation [19]. Exposure to 20 *μ*M imidacloprid (pure compound) and its commercial formulation, Confidor 200 SL, significantly increased DNA strand breaks in leukocytes and micronucleus frequency in peripheral blood lymphocytes, both with and without *in vitro* S9 metabolic activation [18]. Recently, a significant increase in the levels of sister chromatid exchange, micronuclei formation, and chromosomal aberrations was observed in human lymphocytes incubated with 25, 30, 35, or 40 *μ*g/mL of the commercial acetamiprid formulation, Mosetam 20 SP for 24 or 48 h [22]. In laboratory animals, the imidacloprid commercial formulation Confidor and pure imidacloprid compound were positive for micronuclei formation and chromosomal aberrations in Wistar albino rat bone-marrow cells treated with 50 or 100 mg/kg body weight for 90 days [16]. Exposure to 300 mg/kg body weight imidacloprid (Confidor) for 24 h significantly increased micronuclei frequency of in rat bone-marrow cells [19]. *Eisenia fetida *exposed to 0.2 or 0.5 mg/kg imidacloprid in dry soil for 14 days exhibited spermatic malformations. The same species showed a significant dose-dependent increase in DNA damage in coelomocytes exposed to 0.05, 0.1, 0.2, or 0.5 mg/L imidacloprid for 2 h [15]. *Rana N-Hallowell* exposed to 0.05, 0.1, 0.2, 0.5, 8, or 32 mg/L^−1^ imidacloprid (pure compound) for 7 days exhibited a significant increase in the percentage of peripheral erythrocytes with micronuclei and DNA break strands [20].

The molecular mechanisms driving neonicotinoid insecticide genotoxicity are largely unknown. Recent *in vitro* studies have indicated that acetamiprid may induce reactive oxygen species (ROS) generation in bacteria [57]. However, *Yurkat* cells and lymphocytes incubated with imidacloprid did not show increased ROS production [18]. Although these results are inconsistent, we suggest that neonicotinoid insecticides, such as Calypso, Poncho, Gaucho, and Jade, are direct genotoxic agents that could act a source of free radicals or ROS in exposed human cells. ROS, such as superoxide anions (O_2_^●^), hydrogen peroxide (H_2_O_2_), and hydroxyl radicals (OH^−^), are highly reactive with DNA and produce damage, including single- and double-strand DNA breaks and nucleoside modifications. The DNA damage or DNA strand breaks as detected in this study could be considered a kind of lesion potentially premutagenic [58].

Considering that human exposure to pesticides could produce an accumulation of DNA lesions and if the DNA is not repaired could be an initial event in the process of chemical carcionogenesis [47, 48], and the development of tumors may eventually occur [49]. In general, the induction of genotoxicity such as chromosomal and DNA lesions may lead to futher problem of mutagenic and carcinogenic activity [49, 58]. DNA damage is the under cause of mutations leading to cancer [58]. This is in-line with previous epidemiological studies that demonstrated a relationship between pesticides exposure and the occurrence of cancer [49].

In this study, we demonstrated that four commercial neonicotinoid formulations, Jade, Gaucho and Calypso (N-nitroguanidine derivatives), and Poncho (a N-cyano-aminide derivate), directly induce DNA damage in a concentration-dependent manner but independently of their chemical structure. We observed comet formation and production of DNA breaks following exposure to each insecticide. When comparing the genotoxic action of all the tested insecticides, we observed that Jade (granulated imidacloprid) was more genotoxic than Gaucho (emulsion imidacloprid) in human cells *in vitro. *Calypso (thiacloprid) was more genotoxic than Poncho (clothianidin). The chemical composition (e.g., solvents, emulsifiers, dispersion agents, and other additives) of each formulation and the concentration of the active ingredient (i.e., thiacloprid, clothianidin, and imidacloprid) determined the genotoxicity in the human peripheral blood lymphocytes *in vitro.* Genotoxicity was reflected in the differential production of the DNA damage and its effects on cellular viability. Although there were no observed cytotoxic effects following exposure to the neonicotinoid insecticide concentrations used to detect genotoxicity, we did observe that, at high concentrations, all pesticides significantly reduced human lymphocyte viability to 18 × 10^−3^ M Jade, 2.0 × 10^−3^ M Gaucho, 2.0 × 10^−1^ M Calypso, and 1.07 M Poncho. Particularly, we observed that to concentrations of 2.0 × 10^−1^ M Calypso and 1.07 M Poncho produced more than 50% killing of the cells and higher DNA fragmentation which was also reflected by presence apoptotic nuclei or comets without nuclei (“clouds”) and presence of residues of insecticides. Possibly, the chemical formulations of Poncho and Calypso play an important role in tolerance of human peripheral lymphocytes exposed of short time to maximum concentrations for the pesticides. Perhaps, insecticides increased intracellular level of ROS or free radicals in human cells that trigger various damaging process such as apoptosis, the decrease ATP level, inhibiting of intracellular proteins activities and others, and finally metabolic poising with death cell to 30 × 10^−3^ M Jade, 3.3 × 10^−3^ M Gaucho, 2.8 × 10^−1^ M Calypso, and 1.42 M Poncho [59]. Unfortunately, the concentrations used in this study may be difficult to translate to a chronic exposure scenario in humans. Nevertheless, *in vitro* studies are warranted to elucidate the mechanism of toxicity at low-level exposure. In addition, it has reported that using human lymphocytes for the genotoxicity studies could explain the best result for humans.

 Considering the ubiquitous environmental presence of neonicotinoid insecticides, this study provided new information relative to the cytotoxic effects of these agrochemicals. Although further studies investigating the details of cytotoxicity mechanisms are necessary before definitive conclusions can be drawn, our results suggest that these insecticides are risk to organisms. Furthermore, increased DNA damage in human lymphocytes indicates potential genetic hazards posed by commonly used pesticides and emphasizes the need and the importance of protective measures and safety regulations to minimize to exposure.

On the other hand, some studies have documented the presence of residues or metabolic intermediates of imidacloprid (Confidor 200SL), thiacloprid, and clothianidin in fruits, vegetables, soil, and water [60–62]. Therefore, biotransformation of these neonicotinoid insecticides has been studied in plants [63] and mammalian systems [54]. The metabolic pathways of neonicotinoids such as imidacloprid, nitenpyram, thiacloprid, acetamiprid, and dinotefuran have studied in mice [63] and spinach [54]. The major primary metabolic reactions (Phase I metabolism) are hydroxylation, desaturation, dealkylation, sulfoxidation, nitroreduction, catalyzed by microsomal CYP-450 isoenzymes [54, 63, 64]. Cytosolic aldehyde oxidase is a nitroreductase for some neonicotinoids. Phase II metabolism involves methylation, acetylation, and formation of glucoronide, glucoside, amino acid, and sulfate- and glutathione-derived conjugates. Metabolites in some cases contribute to mammalian hepatotoxicity and carcinogenesis [64].

In previous studies, we demonstrated that the *in vivo* vegetal metabolic activation (S10 enzymatic from *Vicia faba* root) of pesticides, such as carbamates, induced significant increases in the frequency of sister chromatid exchange [65–67], DNA strand breaks, and alterations in the cellular proliferation kinetics of the human peripheral lymphocytes [52]. Original compounds and metabolites may pass through the animal digestive tract and can be activated. When the animal and plant are used as food, these compounds could represent a risk to health [68]. On the other hand, when the agrochemical get into food plant, these substances can be objects of further transformations, remain in an unaltered state, or are reactive by human or animal digestive enzymes producing perhaps physiological adverse effects on organisms [68].

 The presence DNA damaged at low concentrations of these neonicotinoid insecticides contributes to the toxicology of these environmental because they are applied on food agricultural fields to higher concentrations (Calypso 22–30 mL/100 L water; Poncho 100 mL/100 L water, Jade 20 Kg/ha, and Gaucho 160 g/Kg seeds) [1] than concentrations assayed in this study and carry up to atmosphere, air, and aquatic systems, and they represent routes of introduction to organisms and are a risk factor on human and animal health.

## 5. Summary

 In summary, our study is the first to show the genotoxic and cytotoxic actions of the neonicotinoid insecticides Calypso, Poncho, Jade, and Gaucho in human peripheral blood lymphocytes *in vitro*. At high concentrations, all pesticides significantly reduced human lymphocyte viability and caused cell death. By comparing the genotoxic and cytotoxic effects of the pesticides, we observed that Jade caused more genotoxicity and cytotoxicity than Gaucho and Calypso and Poncho. These results corroborated that the alkaline comet assay is an excellent and sensitive test to evaluate DNA damage induced by pesticides in human peripheral blood lymphocytes *in vitro.* However, more genotoxic studies with different biological test systems are necessary to confirm that these insecticides are dangerous to animals, including humans, and to clarify the genotoxic and cytotoxic mechanism of neonicotinoid insecticides.

##  Conflict of Interests

Authors declare that there are not conflicts of interest.
